# Report of Surgical Cases in General Hospital, Fayetteville, North Carolina

**Published:** 1864-08

**Authors:** Benjamin F. Fessenden


ART. II.—*Report of Surgical Cases in General Hospital, Fayetteville, North Carolina.* By BENJ. F. FESSENDEN, Surgeon-in-Charge.

John W. Gardener, age 25, private company "F," 24th North Carolina regiment, was wounded at the battle of Fredericksburg, on the 13th December, 1862, by a minie ball entering just above the pubes, passing through the bladder and out through the body of the ischium. He states that he remained two months at a private house in Fredericksburg, with little or no treatment, as the surgeon thought he could not survive the injury. He was then transferred to a hospital at Richmond, where he remained one week, and received a furlough for forty days to go to his home in this (Cumberland) county.

He was admitted into this hospital, November 1st, 1863. Symptoms of great vital depression existing; urine passing through the wounds made by the entrance and exit of the ball; bed sores; excoriations of back and thighs; constipation; loss of appetite; emaciation; the least movement of the body, or an action from the bowels would bring on intense agony.

My first care was to make the patient comfortable, to heal the bed-sores and excoriations: this was accomplished by arranging the bed with an opening lined with oilcloth to drain off the urine, thereby keeping him dry; the following wash to be applied to the excoriated surfaces; borax 2 drachms; tinct. opii 1½ ounces; water 16 ounces; M. Ordered sulph. quinine 32 grains; tinct. chlor. ferri 3 drachms; aquæ 4 ounces; M. Teaspoonful to be given three times daily; brandy 1 oz. three times daily; generous diet. Under this treatment, the patient was rendered much more comfortable—excoriations healed, and appetite improved.

November 10th.—In consultation with Drs. Benjamin W. Robinson and T. D. Haigh, it was decided to examine the wounds and bladder as thoroughly as the present irritable and debilitated condition of the patient would admit. Complete anæsthesia was induced by inhalation of chloroform, a catheter was passed into the bladder, and the presence of a calculus recognized. To be thoroughly satisfied in regard to the diagnosis, the catheter was withdrawn and a sound introduced, which, when brought in contact with the foreign body, removed all doubt as to its nature—the sharp, clear sound of the stone was distinctly heard.

The urine gave a large amount of white deposit, which, upon examination, was found to be the phosphate of lime. To correct the calculous diathesis and improve the general health of the patient was, under existing circumstances, an object of much importance. With this view, was ordered full diet; brandy, 1 ounce after each meal; nitro muriatic acid, 6 gtt, three times daily, properly diluted with sweetened water; laxatives when required. With this treatment, the patient improved in general health, had a good appetite, and had gained flesh.

February 13th, 1864.—*Operation:* present, Drs. Benjamin W. Robinson, T. D. Haigh, J. McRae. H. A. McSwain, W. I. McDuffie, and R. A. Black. By invitation, Dr. Robinson operated. The patient being placed in the usual position and brought fully under the influence of chloroform, a deeply grooved staff was introduced and confided to an assistant. Then a crescentic incision, passing half an inch in front of the anus, divided the tissues intervening to the deep perineal fascia. Considerable hemorrhage from the left superficial perineal artery occurred—not sufficiently troublesome, however, to require attention. An unusually deep perineum caused slight embarrassment in recognizing the position of the staff; but that point being assured, an opening through the fascia and into the membranous portion of the urethra, permitted the introduction to the groove of the beak of a double lithotome, which was passed into the bladder; staff removed, instrument reversed, and the section of the right and left lobes of the prostate, to the extent of ten lines in each, in the oblique diameter, quickly performed. The index finger of the left hand introduced, at once detected the presence of more than one stone, and guided the forceps with which calculi (to the number of four) of very considerable dimensions, were successively and without difficulty extracted. The largest of these weighed an ounce (Troy.) Aggregate weight $2\frac{7}{16}$ ounces.

Chemical composition: phosphate of lime.

The patient was put to bed; no unpleasant effects from the chloroform. Ordered tr. opii, 25 gtt., to be taken at bedtime.

February 14th.—Patient doing well; passed a comfortable night. February 15th.—Water passing through the wound made by the operation, has ceased to flow through the openings made by the ball. February 17th.—Examined urine; found it loaded with a deposit of the phosphate of lime. Ordered nitro muriatic acid, 6 gtt., three times daily; brandy, 1 ounce after each meal; a light, nutritious diet. February 18th to 20th.—Daily visits; patient gradually improving. February 27th.—Passed water by urethra for first time; back wound healed; a small quantity of urine passed by the wound above the pubes. February 28th.—Cauterized the fistulous opening above the pubes. March 9th.—Not entirely healed; was cauterized again. March 15th.—Wound in perineum nearly closed. March 18th.—Had a chill last night, with biliary derangement. Ordered hydr. chlor. mite, 10 grains; rhubarb, 10 grains, M., to be followed after its operation with 10 grains sulph. quinine. March 21st.—No return of chill; doing well. May 2d.—Patient much improved, appetite good, and has gained flesh; bowels regular; all the wounds healed except the one made by exit of the ball, from which there is a slight fistulous discharge, but no urine. The patient is now able to take out-door exercise, with the aid of a walking-cane.

NOTE.—Dr. R. who has operated repeatedly, both by the lateral and bilateral methods with invariable success, inclines to a preference of the latter, as he thinks it affords more facility of manipulation, and the cures are quite as quickly and perfectly attained.

William McNair, private, company "E," 52d regiment N. C. troops, 33 years of age, fair complexion, medium size and spare habit, occupation before the war a farmer, was sent to this hospital, December 17th, 1863, on account of a tumor seated on the soft palate, just in front of the lateral half-arches, cone shape, not circumscribed or movable, but blended with the surrounding tissue, presenting a dark-red appearance, with a number of blood vessels ramifying pretty freely over its surface. The size of the tumor precluded possibility of the patient taking any nourishment, except in a fluid state. He states that it made its appearance about six years ago, that its growth had been gradual, accompanied occasionally with sharp, darting pains, but generally a dull, aching sensation. Ordered sulph. quinine, 32 grains; tinct. chlor. ferri, 3 drachms; aqua, 4 ounces, M.—a teaspoonful to be taken three times daily. This treatment, in conjunction with a generous diet, was continued until February 2d, 1864, at which time his appetite was good, and general health much improved in every respect.

On consultation with Dr. James McRae, it was thought advisable to operate at once. From the vascular appearance of the tumor and the difficulty of suppressing hemorrhage, owing to its location, it presented an obvious case for the use of the ecraseur. I am indebted to my friend, Dr. Edwin Anderson, of Wilmington, for the use of that instrument.

February 2d, 1864.—*Operation:* The patient was placed in a chair, his head supported by an assistant, the loop of the chain of the ecraseur was passed over the tumor and carried well up to its base. Owing to its cone shape, it was, with difficulty, retained in that position; this was overcome by the index fingers of an assistant being inserted at the angles of the mouth, and retaining the chain well up at the base of the tumor. This method was thought prfeerable to transfixing the tumor with needles; the chain being adjusted, it was strongly tightened, causing the pedunculation of the tumor; then, by a gradual and steady turn of the screw, the separation of the mass was accomplished. Very little hemorrhage followed. The patient was put to bed, and directed to use a weak solution of alum water as a gargle. The contents of the tumor presented a brain-like substance, exhaling a very fœtid odor. The microscope, to which a portion of it was subjected, revealed the rounded and caudate nucliferous cells, (true characteristics of malignant disease.)

February 3d.—Patient passed a disagreeable night, owing to the offensive discharge from the wound. Ordered creosote, ½ drachm; gum arabic, 2 drachms; aquæ, 8 ounces, M., to be used as a gargle every three or four hours. February 4th.—Wound sloughing; very offensive; continued creosote gargle. February 5th.—Slough separated, leaving a healthy, granulated surface. February 6th and 7th.—Rested well at night; wound presents a healthy appearance. April 13th.—Continued to improve; wound has healed perfectly; cicatrix remains sound; has been doing light work about the hospital for the last three weeks; no indications of the return of the disease; returned to duty.

NOTE.—This man states that he had an uncle who died from cancer of the tongue.

The above case presents some well-marked features of medullary sarcoma, but is lacking in others equally characteristic of the disease—time alone can testify as to its true character.

